# Favorable, arduous or fatal postoperative pathway within 90 days of lung transplantation

**DOI:** 10.1186/s12890-022-02120-w

**Published:** 2022-08-27

**Authors:** Alexy Tran-Dinh, Donia Bouzid, Adnan El Kalai, Enora Atchade, Sébastien Tanaka, Brice Lortat-Jacob, Sylvain Jean-Baptiste, Nathalie Zappella, Sandrine Boudinet, Yves Castier, Hervé Mal, Pierre Mordant, Jonathan Messika, Philippe Montravers

**Affiliations:** 1 Université Paris Cité, AP-HP, Hôpital Bichat Claude Bernard, Anesthésie-Réanimation, Paris, France; 2 INSERM UMR 1148 LVTS, Université Paris Cité, Paris, France; 3 Université Paris Cité, AP-HP, Hôpital Bichat Claude Bernard, Service des Urgences, Paris, France; 4 INSERM UMR 1137 IAME, Paris, France; 5 INSERM UMR 1188 DéTROI, Université de la Réunion, Saint-Denis de la Réunion, France; 6 Université Paris Cité, AP-HP, Hôpital Bichat Claude Bernard, Service de Chirurgie Vasculaire, Thoracique et Transplantation Pulmonaire, Paris, France; 7 INSERM UMR 1152 PHERE, Université Paris Cité, Paris, France; 8 Université Paris Cité, AP-HP, Hôpital Bichat Claude Bernard, Pneumologie B et Transplantation Pulmonaire, Paris, France; 9 Paris Transplant Group, Paris, France

**Keywords:** Lung transplantation, Intensive care unit, Mortality, Morbidity, Intraoperative ECMO

## Abstract

**Introduction:**

The maximum gain in quality of life after lung transplantation (LT) is expected between six months and one year after LT, as the occurrence of chronic lung allograft dysfunction may mask the beneficial effects beyond one year. Thus, the postoperative period could be the cornerstone of graft success. We sought to describe the factors present before postoperative admission to the ICU and associated with favorable, arduous or fatal pathway within 90 days of LT.

**Materials and methods:**

We conducted a retrospective single-center study between January 2015 and December 2020. Using multinomial regression, we assessed the demographic, preoperative and intraoperative characteristics of patients associated with favorable (duration of postoperative mechanical ventilation < 3 days and alive at Day 90), arduous (duration of postoperative mechanical ventilation ≥ 3 days and alive at Day 90) or fatal (dead at Day 90) pathway within 90 days of LT.

**Results:**

A total of 269 lung transplant patients were analyzed. Maximum graft cold ischemic time ≥ 6 h and intraoperative blood transfusion ≥ 3 packed red blood cells were associated with arduous and fatal pathway at Day 90, whereas intraoperative ECMO was strongly associated with fatal pathway.

**Conclusion:**

No patient demographics influenced the postoperative pathway at Day 90. Only extrinsic factors involving graft ischemia time, intraoperative transfusion, and intraoperative ECMO determined early postoperative pathway.

**Supplementary Information:**

The online version contains supplementary material available at 10.1186/s12890-022-02120-w.

## Introduction

Lung transplantation (LT) is currently recognized as a life-saving therapy for patients with end-stage lung disease. LT represents more than 4000 procedures per year in selected patients for whom optimal medical treatment is no longer sufficient to maintain quality of life or short-term survival [1, 2]. However, the overall median survival of 6.7 years is still lower than that of any of the other solid organ transplants and is impacted by early deaths mainly related to graft dysfunction, infections and cardiovascular complications [3].

The maximum gain in quality of life is expected between six months and one year after LT, as the occurrence of chronic lung allograft dysfunction may mask the beneficial effects beyond one year [4]. Thus, the postoperative period could be the cornerstone influencing patient outcome and transplant success. Unfortunately, it remains very difficult to predict the patient's early postoperative prognosis. Basically and arbitrarily, the early pathway of patients after LT could follow three trajectories: favorable, arduous and fatal.

Our study sought to identify factors present before postoperative admission to the intensive care unit (ICU) and associated with favorable, arduous and fatal pathway within 90 days of LT.

## Materials and methods

### Study design

We conducted a retrospective single-center study that included all patients who underwent LT at our institution between January 2015 and December 2020.

#### Definition of favorable, arduous and fatal pathway within 90 days of LT

No definition exists to define favorable and arduous pathway within 90 days of LT. Therefore, we arbitrarily define favorable pathway as lung transplant patients with a duration of postoperative mechanical ventilation < 3 days and alive at Day 90, and arduous pathway as a duration of postoperative mechanical ventilation ≥ 3 days and alive at Day 90. Fatal pathway was defined as death within 90 days of LT. Three days of mechanical ventilation is the median duration in our cohort.

#### Assessment of factors present before postoperative admission to the ICU and associated with favorable, arduous or fatal pathway within 90 days of LT

Demographic, preoperative and intraoperative characteristics of patients were compared between favorable arduous and fatal pathway within 90 days of LT.

#### Ethics

All the experiment has been performed in accordance with the Declaration of Helsinki. The study was approved by the ethics committee CEERB Paris Nord, which waived the need for signed informed consent (Institutional Review Board -IRB 00,006,477- University of Paris, AP-HP.Nord. No organs/tissues were procured from prisoners and organs/tissues were procured exclusively from French hospitals.

### Data collection

We recorded the following data: (1) Demographic and preoperative characteristics of the patients (age, sex, body mass index (BMI), primary diagnosis of chronic pulmonary disease, Cytomegalovirus mismatch (Donor + /Recipient-), past medical history of diabetes and ischemic heart disease with angioplasty and/or coronary stent, high-emergency LT, extracorporeal membrane oxygenation (ECMO) as a bridge to transplant, mean pulmonary arterial pressure (mPAP) measured by a right heart catheterization at listing; (2) Intraoperative characteristics (type of LT, i.e., single or bilateral, maximum graft cold ischemia time, intraoperative blood transfusion, intraoperative ECMO and thoracic epidural analgesia); (3) specific lung transplant complications (grade 3 primary graft dysfunction (PGD) as defined by the ISHLT consensus [5], bronchial anastomosis dehiscence, acute cellular rejection confirmed by histopathological evidence after transbronchial lung biopsies performed only in cases of suspicion and not systematically [6], and definite, probable or possible antibody-mediated rejection, according to Levine et al. [7], with the need for plasmapheresis, (4) ICU stay characteristics (simplified acute physiology score II (SAPS II) and sequential organ failure assessment (SOFA) score at admission, acute kidney injury stage 3 of KDIGO, renal replacement therapy, duration of mechanical ventilation, duration of norepinephrine support, ECMO in ICU, tracheostomy, ICU length of stay); and (6) mortality rates during the postoperative ICU stay and at Day 90.

### Perioperative management

Surgical transplantation procedures and perioperative care, including postoperative management, were standardized for all patients according to our local protocol that was published elsewhere [8, 8]. The immunosuppressive regimen included mycophenolate mofetil, corticosteroids and tacrolimus. Perioperative antibiotics were routinely administered for 48 h after LT. Cefazolin (or the antibiotic that was administered to the donor before LT) was the standard antibiotic prophylaxis. In the postoperative period, antibiotic therapy was adapted to the microbiological cultures obtained from the bronchoalveolar lavage performed systematically in postoperative admission to the ICU, and then performed according to clinical suspicion of pneumonia [10].

### Statistical analysis

Baseline characteristics within each group were described with numbers and percentages for categorical variables and medians and interquartile ranges (IQR) for quantitative variables. We compared the characteristics of the three different pathways (favorable, arduous or fatal) using the Kruskal–Wallis test for quantitative variables and the χ2 or Fisher test for the categorical variables. We used multinomial regression as a multivariable analysis to assess the factors associated with those pathways (Results are represented in relative risks with their 95% confidence intervals (CI) [11]. Kaplan Meier survival curves were constructed for the 365-day period after lung the transplantation. Missing data were not replaced in the final dataset. The *P* values corresponded to the Wald statistic, and a threshold of 0.05 was used for statistical significance. Statistical analysis and data management were performed using Stata/IC^®^15.

## Results

**Fig. 1:**
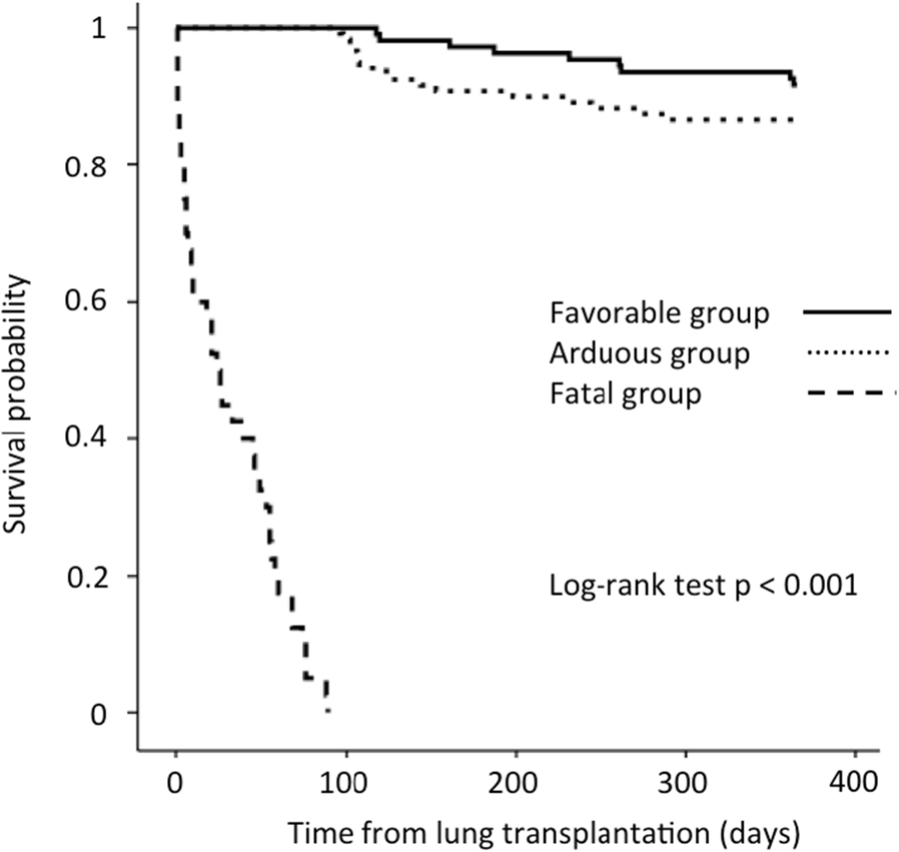
Kaplan–Meier curve at 365 days for patients with favorable, arduous or fatal pathway

During the study period, 269 patients underwent LT. Most of the patients were male (64.3%), with a median (IQR) age of 57 (51–62) years. The main etiologies for LT were interstitial lung diseases (ILD) (48.7%) and chronic obstructive disease pulmonary disease (COPD) (36.4%). Patients had favorable (n = 109, 40.5%), arduous (n = 120, 44.6%) or fatal (n = 40, 14.9%) pathway within 90 days of LT (Table 1), with a median (IQR) hospital length of stay of 37 [31–50] days, 71 [49–112] days and 23 [6–55] days, respectively. The overall survival of the cohort was 91.8% at 30 days, 85.1% at 90 days and 76.2% at one year. Kaplan–Meier curve for survival at 365 days for patients with favorable, arduous or fatal pathway is displayed in the Fig. 1.

**Table 1:** Univariate analysis of factors present before posttransplant ICU admission and associated with favorable, arduous or fatal pathway within 90 days

	LT recipients (n = 269)	Favorable (n = 109)	Arduous (n = 120)	Fatal (n = 40)	Univariate analysis *p* value
*Recipient characteristics*					
Age ≥ 60 years, n (%)	93 (34.6)	48 (44.0)	33 (27.5)	12 (30.0)	0.03
Male sex, n (%)	173 (64.3)	65 (59.6)	81 (67.5)	27 (67.5)	0.44
Body mass index ≥ 30 kg/m^2^, n (%)	35 (13.0)	9 (8.3)	21 (17.5)	5 (12.5)	0.12
Primary diagnosis, n (%)					0.05
COPD	98 (36.4)	49 (45.0)	67 (55.8)	19 (47.5)	0.05
ILD	131 (48.7)	44 (40.4)	38 (31.2)	11 (27.5)	0.06
Others	41 (15.2)	16 (14.7)	15 (12.5)	10 (25.0)	0.17
Cytomegalovirus mismatch (Donor + /Recipient-), n (%)	55 (20.5)	27 (24.8)	20 (16.7)	8 (20)	0.31
Waiting list time, days	69 [23–182]	61 [23–156]	72 [25–194]	129 [8–209]	0.69
High-emergency LT, n (%)	49 (18.2)	10 (9.2)	26 (21.7)	13 (32.5)	0.002
Preoperative ECMO, n (%)	19 (7.1)	1 (0.92)	12 (10.0)	6 (15.0)	0.001
Pretransplant diabetes, n (%)	28 (10.4)	12 (11.0)	13 (10.8)	3 (7.5)	0.93
Pretransplant ischemic heart disease with coronary angioplasty and/or stent, n (%)	11 (4.1)	7 (6.4)	2 (1.7)	2 (5.0)	0.15
Pretransplant mPAP ≥ 25 mmHg, n (%)	136 (55.3)	60 (58.3)	54 (49.5)	22 (64.7)	0.23
% predicted 6 MWT, (%)	43 [28–55]	45 [31–58]	41 [28–54]	43 [23–58]	0.18
*Lung transplant surgery*					
Type of LT, n (%)					0.11
Single LT	87 (32.3)	43 (39.5)	32 (26.7)	12 (30.0)	
Double LT	182 (67.7)	66 (60.6)	88 (73.3)	28 (70.0)	
Maximum graft ischemia time ≥ 6 h, n (%)	114 (42.3)	35 (32.7)	57 (48.7)	22 (61.1)	0.005
Intraoperative blood transfusion (≥ 3 PRBCs), n (%)	127 (47.2)	34 (31.2)	66 (55.0)	27 (67.5)	< 0.001
Intraoperative ECMO, n (%)	190 (70.6)	62 (56.9)	90 (75.0)	38 (95.0)	< 0.001
Thoracic epidural analgesia, n (%)	145 (53.9)	65 (59.6)	61 (50.8)	19 (47.5)	0.28

Quantitative variables are expressed as medians and interquartiles. Categorical variables are expressed as numbers and percentages

*COPD* Chronic obstructive pulmonary disease, *ILD* interstitial lung disease, *LT* lung transplantation, *PAP* pulmonary artery pressure, *6 MWT* six-minute walk test, *PRBC* packed red blood cell, *ECMO* extracorporeal membrane oxygenation

Patient characteristics before admission to the postoperative intensive care unit (ICU) and postoperative outcomes after LT are presented in Tables 1 and 2, respectively.

**Table 2:** Postoperative ICU outcomes and lung graft complications

	LT recipients (n = 269)	Favorable (n = 109)	Arduous (n = 120)	Fatal (n = 40)	*p* value
*Postoperative ICU stay*					
SOFA score at admission	7 [6–9]	7 [5–8]	8 [6–9]	9 [8–10]	< 0.001
SAPS II at admission	43 [38–50]	41 [36–46]	46 [40–54]	50 [43–70]	< 0.001
Duration of norepinephrine support, days	2 [1–4]	1 [1]	1 [3–6]	3 [3–9]	< 0.001
Duration of mechanical ventilation, days	3 [1–16]	1 [1]	12 [3–30]	8 [2–32]	< 0.001
Acute kidney injury stage 3 of KDIGO	38 (14.1)	0 (0)	15 (12.5)	23 (57.5)	< 0.001
Renal replacement therapy, n (%)	30 (11.2)	0 (0)	9 (7.5)	21 (52.5)	< 0.001
ECMO in ICU, n (%)	76 (28.3)	3 (2.8)	48 (40)	25 (62.5)	< 0.001
ICU length of stay, days	17 [3, 10–30]	11 [8–14]	26 [17–50]	18 [6–41]	< 0.001
Tracheotomy, n (%)	66 (24.5)	1 (0.9)	55 (45.8)	10 (25)	< 0.001
Time to tracheostomy, days	16 (13–19)	14	16 [3, 13–17]	15 [3, 12–16]	0.76
ICU mortality, n (%)	39 (14.5)	0 (0)	7 (5.8)	32 (80)	< 0.001
Time to ICU mortality, days	21 [6–76]	NA	107 [102–124]	36 [10–90]	< 0.001
*Graft complications within 90 days*					
Grade 3 primary graft dysfunction, n (%)	48 (17.8)	0 (0)	36 (30)	12 (30)	< 0.001
Antibody-mediated rejection, n (%)	43 (16.0)	13 (11.9)	23 (19.2)	7 (17.5)	0.3
Acute cellular rejection, n (%)	34 (12.6)	12 (11)	16 (13.3)	6 (15)	0.7
Bronchial anastomosis dehiscence, n (%)	43 (16.0)	7 (6.4)	25 (20.8)	11 (27.5)	0.01

Quantitative variables are expressed as medians and interquartiles. Categorical variables are expressed as numbers and percentages

*ICU* intensive care unit, *SOFA* sequential organ failure assessment, *SAPS* II simplified acute physiology score II, *KDIGO* kidney disease improving global outcomes, *NA* not applicable

### Factors present before postoperative admission to the ICU and associated with favorable, arduous or fatal pathway within 90 days of LT

In univariate analysis, age ≥ 60 years, high emergency LT, ECMO as a bridge to transplant, maximum graft cold ischemic time ≥ 6 h, intraoperative blood transfusion ≥ 3 PRBCs and intraoperative ECMO were factors associated with a non-favorable pathway within 90 days (Table 1).

After multinomial regression, a maximum graft cold ischemic time ≥ 6 h and intraoperative blood transfusion ≥ 3 PRBCs were independently associated with arduous or fatal pathway within 90 days, whereas intraoperative ECMO was strongly and independently associated with fatal pathway only (Table 3).

**Table 3:** Multinomial logistic regression for risk factors influencing 90-day pathway after LT

	Relative risk	95%CI	*p* value
Favorable pathway	Reference		
*Arduous pathway*			
Intraoperative ECMO	1.83	0.99–3.40	0.12
Maximum graft ischemia time ≥ 6 h	1.91	1.08–3.37	0.03
Intraoperative blood transfusion (≥ 3 PRBC)	2.20	1.22–3.97	0.009
*Fatal pathway*			
Intraoperative ECMO	10.22	2.23–46.81	0.003
Maximum graft ischemia time ≥ 6 h	3.32	1.45–7.58	0.004
Intraoperative blood transfusion (≥ 3 PRBC)	2.49	1.05–5.86	0.04

*ECMO* Extracorporeal membrane oxygenation, *PRBC* packed red blood cell

### Factors associated with intraoperative ECMO, graft cold ischemia time ≥ 6 h and intraoperative blood transfusion ≥ 3 PRBCs

Intraoperative ECMO was associated with ILD, high emergency LT, ECMO as a bridge to transplant, pretransplant mPAP ≥ 25 mmHg and intraoperative blood transfusion ≥ 3 PRBCs. Interstitial lung disease and pretransplant mPAP ≥ 25 mmHg remained independently associated with intraoperative ECMO after multivariate analysis (Additional file 1: Table S1).

Graft cold ischemia time ≥ 6 h was associated with age ≥ 60 years (protective factor), other primary diagnoses than COPD or ILD, single LT (protective factor, as expected) and intraoperative blood transfusion ≥ 3 PRBCs. After multivariate analysis, age ≥ 60 years was as an independent protective factor for graft cold ischemia time ≥ 6 h (Additional file 1: Table S2).

Intraoperative blood transfusion ≥ 3 PRBCs was associated with age ≥ 60 years (protective factor), other primary diagnoses than COPD or ILD, cytomegalovirus mismatch (protective factor), high emergency LT, ECMO as a bridge to transplant, double LT, graft cold ischemia time ≥ 6 h and thoracic epidural analgesia (protective factor). After multivariate analysis, cytomegalovirus mismatch and thoracic epidural analgesia were independent protective factors for intraoperative blood transfusion ≥ 3 PRBCs, whereas high emergency LT and bilateral LT were independent risk factors (Additional file 1: Table S3: Factors associated with intraoperativr ECMO, graft cold ischemia time  ≥ 6 hours and intraoperative blood transfusion ≥ 3 PRBCs ).

## Discussion

We explored for the first time, to our knowledge, the risk factors present before postoperative ICU admission and associated with three trajectories of pathway, favorable, arduous or fatal, within 90 days after LT. Intraoperative ECMO, graft cold ischemic time ≥ 6 h and intraoperative blood transfusion ≥ 3 PRBCs were independent risk factors for arduous or fatal pathway at Day 90.

Intraoperative ECMO was strongly associated with a comorbid postoperative stay in the ICU, grade 3 PGD and 90-day mortality. The impact of intraoperative ECMO on 90-day survival has been poorly studied. Zhang et al. recently showed that patients with intraoperative ECMO were more likely to have an increased 3-month mortality rate compared with those without ECMO, although it did not reach statistical significance (27.3 vs. 17.2%, *p* = 0.25) [12]. The largest studies of perioperative ECMO use have primarily evaluated patient survival at one year and beyond, with contradictory results. Ius et al. showed higher in-hospital mortality rates [13] and higher 3-, 5- and 8-year mortality rates for patients requiring intraoperative ECMO [14], although ECMO was not selected as an independent risk factor. In contrast, Hoetzenecker et al. observed improved 1-, 3-, and 5-year survival rates compared with non-ECMO patients [15]. It should be noted that central cannulation was predominant, except for patients using peripheral ECMO as a bridge to transplant.

The high rate of intraoperative ECMO (70%) in our cohort and the poor prognosis of patients under ECMO could be explained by their significant comorbidities. Forty-nine patients required preoperative ECMO and all were transplanted with intraoperative ECMO using the high-emergency procedure. Therefore, candidates for high-emergency transplantation requiring preoperative ECMO should be carefully selected. The median (IQR) and mean ± SD age were 56 (50–62) years and 54 ± 11 years, respectively, compared to 48 (31–55) years in the study of Ius et al. [13] and 45.2 ± 16.2 years in the study of Hoetzenecker et al. [15]. In a retrospective analysis of 8363 patients from the UNOS database, Weiss et al. showed that older patients had an increased risk of death after LT [16]. Pulmonary fibrosis, a well-known risk factor for intraoperative ECMO [12, 12] and for higher posttransplant mortality [3], represented 58% of our transplant recipients, compared to 30% in the two studies mentioned above [13, 13]. Single LT accounted for 31.5% of the procedures, whereas it was only 3% for the study of Ius et al. [13] and the study of Hoetzenecker et al. exclusively focused on bilateral LT [15]. Single LT has previously been identified as a risk factor for intraoperative ECMO [13] and overall poorer survival rates compared with double LT [17]. In addition, 66.8% of patients with intraoperative ECMO were male, compared to 54% [13] and 48% [15] in the other studies. Male sex was consistently associated with a higher mortality rate after LT [18–21].

In our cohort, the vast majority of intraoperative ECMO was unplanned, except for patients on ECMO bridging before LT. Our practice is to implant ECMO "on demand” when irreversible hemodynamic and/or respiratory instability is deemed irreversible by the practitioners in charge of the patient occurs during surgery, especially when clamping the pulmonary artery branch. However, the impact of intraoperative ECMO on survival does not appear to be related to implantation strategies, as Ius et al. observed no difference in survival between a priori and on-demand strategies [13]. The rate of prolonged ECMO during the postoperative course in the ICU was 40% and was similar to their rates of 41% [13] and 36% [15].

We identified a primary diagnosis of ILD and pretransplant mPAP ≥ 25 mmHg as risk factors for intraoperative ECMO, as previously reported [12, 12]. The association of intraoperative ECMO with a greater risk of grade 3 PGD confirmed what had previously been observed [14].

The impact of graft cold ischemic time on graft failure and survival remains under debate. Prolonged ischemia ≥ 6 h was an independent risk factor for arduous and fatal pathway within 90 days and was likely to be associated with more reports of grade 3 PGD, although the difference did not reach statistical significance. Some previous studies have reported similar results [22, 22], while others have found no association [24–26]. However, it was suggested that the cold ischemia time could have a greater impact for the most fragile patients [26]. We identified recipient age ≥ 60 years, a marker of frailty, as an independent factor associated with prolonged ischemia ≥ 6 h.

Intraoperative blood transfusion ≥ 3 PRBCs was independently associated with arduous and fatal pathway, with more reports of comorbidities during postoperative ICU stay and more reports of grade 3 PGD. Blood loss during surgery has been consistently associated with more postoperative complications, but the impact on mortality remains unclear [27, 27]. Factors associated with high PRBC transfusion during surgery were high-emergency LT and double LT. High-emergency LT is a national prioritization system for the most severe patients with fibrosis, cystic fibrosis or pulmonary hypertension that was introduced in France in 2007. As a different method than the Lung Allocation Score [29], the allocation rules in France are developed by the Agence de la Biomédecine in collaboration with the transplant community, and this is the responsibility of a lung transplant team that selects the recipient it believes will benefit most from the allograft [30]. The Agence de la Biomédecine develops the allocation rules in France in collaboration with the transplant community. Before September 2020, the lung transplant allocation system was based on national allocation for patients with high-emergency status and local, regional, and then national allocation for elective patients. However, this transplant allocation model has generated significant geographic disparities across transplant centers, with an average of grafts offered per candidate ranging from 1.4 to 5.2. Thus, as of 8 September 2020, a new system was implemented that restricted the local allocation according to the supply/demand ratio, eliminating regional sharing and increasing national sharing. The supply/demand ratio was defined as the ratio of the number of lungs recovered in the local allocation unit to transplants performed in the center [31]. Patients can be candidates for the high-emergency waiting list if they need mechanical ventilation or if they are at risk of undergoing mechanical ventilation with an oxygen dependency greater than 12 L/min associated with an SpO2 < 90% despite maximal treatment and in the absence of a reversible cause. The presence of severe organ failure or uncontrolled sepsis contraindicates access to this emergency procedure [32]. Overall, this indication is assessed by independent and anonymous experts designed by the French Biomedicine agency. Patients on a high emergency waiting list often require ECMO as a bridge to transplant, a condition that has been associated with blood loss during LT [28]. In contrast, patients who received thoracic epidural analgesia were less likely to receive a massive transfusion. We hypothesized that epidural anesthesia, by providing sympathetic blockade, may reduce peripheral venous bleeding mediated by venous hypotension resulting from decreased peripheral resistance, while vital organ perfusion is maintained by sustaining cardiac output through norepinephrine and fluid management. Hypotensive epidural anesthesia has been shown to reduce blood loss in urological surgery [33].

This study has several major limitations, which are its single-center and retrospective nature. The external validity of our results must be interpreted with caution, as these observations depend on the etiologies of LT, the selection of candidate patients, and their management, all of which may vary from one center to another.

## Conclusion

We identified and deciphered three independent risk factors present prior to ICU admission after LT and associated with arduous or fatal pathway within 90 days. Patient candidates aged ≥ 60 years with a primary diagnosis of ILD, preoperative pulmonary hypertension, undergoing a high emergency bilateral LT or LT, should be warned of an expected hemorrhagic surgery requiring ECMO, with an increased risk of unfavorable pathway.

## Supplementary Information

**Additional file 1**.** Table S1**. Factors associated with intraoperative ECMO.** Table S2**. Factors associated with graft cold ischemia time ≥ 6 hours.** Table S3**. Factors associated with intraoperative blood transfusion ≥ 3 PRBCs.

## Data Availability

The datasets used and/or analyzed during the current study are available from the corresponding author on reasonable request.

## Abbreviations

BMI: Body mass index
COPD: Chronic obstructive pulmonary disease
ECMO: Extracorporeal membrane oxygenation
ICU: Intensive care unit
ILD: Interstitial lung diseases
LT: Lung transplantation
SAPS II: Simplified acute physiology score II
SOFA: Sequential organ failure assessment
