# Supplementary material for: Favorable, arduous or fatal postoperative pathway within 90 days of lung transplantation
Source: BMC Pulm Med. 2022 Aug 27;22:326. doi: 10.1186/s12890-022-02120-w (PMC9420258; doi:10.1186/s12890-022-02120-w)
Supplement: Supplementary file 1 — Additional file 1. Table S1. Factors associated with intraoperative ECMO. Table S2. Factors associated with graft cold ischemia time ≥ 6 hours. Table S3. Factors associated with intraoperative blood transfusion ≥ 3 PRBCs. [file 12890_2022_2120_MOESM1_ESM.docx]

**Table S1: Factors associated with intraoperative ECMO**

|  | **Intraoperative ECMO**  **(n=190)** | **No intraoperative ECMO**  **(n=79)** | **Univariate,**  **p value** | **Multivariate**  **OR [95%CI],**  **p value** |
| --- | --- | --- | --- | --- |
| **Recipient characteristics** |  |  |  |  |
| Age ≥ 60 years, n (%) | 67 (35.3) | 26 (32.9) | 0.78 |  |
| Male sex, n (%) | 127 (66.8) | 46 (58.2) | 0.21 |  |
| Body mass index ≥ 30 kg/m^2^, n (%) | 8 (10.1) | 27 (14.2) | 0.43 |  |
| Primary diagnosis, n (%) |  |  | <0.001 |  |
| COPD | 50 (26.3) | 48 (60.8) | <0.001 |  |
| ILD | 110 (57.9) | 20 (25.3) | <0.001 | 5.05 [2.70-9.44], p<0.0001 |
| Others | 30 (15.8) | 11 (13.9) | 0.85 |  |
| Cytomegalovirus mismatch (Donor+/Recipient-), n (%) | 38 (20.0) | 17 (21.5) | 0.87 |  |
| Waiting list time, days, median (IQR) | 69 (23-182) | 75 (34-235) | 0.07 |  |
| High-emergency lung transplantation, n (%) | 48 (25.3) | 1 (1.3) | <0.001 |  |
| Preoperative ECMO, n (%) | 19 (10.0) | 0 (0.0) | 0.001 |  |
| Pretransplant diabetes | 23 (12.1) | 5 (6.3) | 0.19 |  |
| Pretransplant ischemic heart disease with coronary angioplasty and/or stent, n (%) | 9 (4.74) | 2 (2.5) | 0.52 |  |
| Pretransplant mPAP ≥ 25 mmHg, n (%) | 103 (61.3) | 33 (42.3) | 0.006 | 2.78 [1.53-5.07],  P = 0.001 |
| **Lung transplantation** |  |  |  |  |
| Type of LT, n (%) |  |  | 0.67 |  |
| Single LT | 60 (31.6) | 27 (34.2) |  |  |
| Double LT | 130 (68.4) | 52 (65.8) |  |  |
| Intraoperative blood transfusion  (≥ 3 PRBCs), n (%) | 112 (59.0) | 15 (19.0) | < 0.001 |  |
| Maximum graft ischemia time > 6 hours, n (%) | 80 (43.7) | 34 (44.2) | 1 |  |
| Thoracic epidural analgesia, n (%) | 100 (52.6) | 45 (57.0) | 0.59 |  |

**Abbreviations:** COPD: chronic obstructive pulmonary disease, ILD: interstitial lung disease, LT: lung transplantation, PAP: pulmonary artery pressure, ECMO: extracorporeal membrane oxygenation, PRBC: packed red blood cell

**Table S2: Factors associated with graft cold ischemia time ≥ 6 hours**

|  | **Maximum graft ischemia time ≥ 6 hours**  **(n=114)** | **Maximum graft ischemia time < 6 hours**  **(n=146)** | **Univariate,**  **p value** | **Multivariate**  **OR [95%CI],**  **p value** |
| --- | --- | --- | --- | --- |
| **Recipient characteristics** |  |  |  |  |
| Age ≥ 60 years, n (%) | 26 (22.8) | 64 (43.8) | <0.001 | 0.39 [0.22-0.65],  p < 0.001 |
| Male sex, n (%) | 76 (66.7) | 92 (63.0) | 0.60 |  |
| Body mass index ≥ 30 kg/m^2^, n (%) | 15 (13.2) | 19 (13.0) | 1 |  |
| Primary diagnosis, n (%) |  |  | 0.04 |  |
| COPD | 38 (33.3) | 57 (39.0) | 0.37 |  |
| ILD | 52 (45.6) | 75 (51.2) | 0.46 |  |
| Others | 24 (21.1) | 14 (9.6) | 0.01 |  |
| Cytomegalovirus mismatch (Donor+/Recipient-), n (%) | 21 (18.4) | 33 (22.6) | 0.44 |  |
| Waiting list time, days, median (IQR) | 73 (25-182) | 69 (23-182) | 0.97 |  |
| High-emergency lung transplantation, n (%) | 21 (18.4) | 25 (17.1) | 0.87 |  |
| Preoperative ECMO, n (%) | 10 (8.8) | 9 (5.5) | 0.33 |  |
| Pretransplant diabetes, n (%) | 8 (7.0) | 19 (13.0) | 0.15 |  |
| Pretransplant ischemic heart disease with coronary angioplasty and/or stent, n (%) | 4 (3.5) | 7 (4.8) | 0.76 |  |
| Pretransplant mPAP ≥ 25 mmHg, n (%) | 53 (51.5) | 78 (56.9) | 0.43 |  |
| **Lung transplantation** |  |  |  |  |
| Type of LT, n (%) |  |  | <0.0001 |  |
| Single LT | 17 (14.9) | 67 (45.9) |  |  |
| Double LT | 97 (85.1) | 79 (54.1) |  |  |
| Intraoperative blood transfusion  (≥ 3 PRBCs), n (%) | 62 (50.8) | 61 (49.2) | 0.03 |  |
| Thoracic epidural analgesia, n (%) | 62 (54.4) | 78 (53.4) | 0.90 |  |

**Abbreviations:** COPD: chronic obstructive pulmonary disease, ILD: interstitial lung disease, LT: lung transplantation, PAP: pulmonary artery pressure, ECMO: extracorporeal membrane oxygenation, PRBC: packed red blood cell

**Table S3: Factors associated with intraoperative blood transfusion ≥ 3 PRBCs**

|  | **Intraoperative blood transfusion**  **≥ 3 PRBCs**  **(n=127)** | **Intraoperative blood transfusion**  **≤ 2 PRBCs**  **(n=142)** | **Univariate**  **p value** | **Multivariate**  **OR [95%CI,],**  **p value** |
| --- | --- | --- | --- | --- |
| **Recipient characteristics** |  |  |  |  |
| Age ≥ 60 years, n (%) | 31 (24.4) | 62 (43.7) | 0.001 |  |
| Male sex, n (%) | 75 (59.1) | 98 (69.0) | 0.10 |  |
| Body mass index ≥ 30 kg/m^2^, n (%) | 17 (13.4) | 18 (12.7) | 1 |  |
| Primary diagnosis, n (%) |  |  | 0.004 |  |
| COPD | 37 (29.1) | 61 (43.0) | 0.02 |  |
| ILD | 62 (48.8) | 68 (47.9) | 1 |  |
| Others | 28 (22.1) | 13 (9.2) | 0.004 |  |
| Cytomegalovirus mismatch (Donor+/Recipient-), n (%) | 18 (14.2) | 36 (26.1) | 0.02 | 0.43 [0.22-0.83], p=0.01 |
| Waiting list time, days, median (IQR) | 81 (26-192) | 66 (21-179) | 0.51 |  |
| High-emergency lung transplantation, n (%) | 32 (25.2) | 17 (12.0) | 0.007 | 2.39 [1.15-4.91],  p=0.04 |
| Preoperative ECMO, n (%) | 15 (11.8) | 1 (0.7) | <0.001 |  |
| Pretransplant diabetes, n (%) | 15 (11.8) | 13 (9.2) | 0.55 |  |
| Pretransplant ischemic heart disease with coronary angioplasty and/or stent, n (%) | 3 (2.4) | 8 (5.6) | 0.23 |  |
| Pretransplant mPAP ≥ 25 mmHg, n (%) | 66 (59.5) | 70 (51.9) | 0.25 |  |
| **Lung transplantation** |  |  |  |  |
| Type of LT, n (%) |  |  | <0.001 | 3.8 [2.13-6.78], p<0.001 |
| Single LT | 24 (18.9) | 63 (44.4) |  |  |
| Double LT | 103 (81.1) | 79 (55.6) |  |  |
| Maximum graft ischemia time > 6 hours, n (%) | 62 (50.8) | 52 (37.7) | 0.03 |  |
| Thoracic epidural analgesia, n (%) | 56 (44.1) | 89 (62.7) | 0.003 | 0.51 [0.30-0.87], p=0.01 |

**Abbreviations:** COPD: chronic obstructive pulmonary disease, ILD: interstitial lung disease, LT: lung transplantation, PAP: pulmonary artery pressure, ECMO: extracorporeal membrane oxygenation, PRBC: packed red blood cell
